# Pathway choice in DNA double strand break repair: observations of a balancing act

**DOI:** 10.1186/2041-9414-3-9

**Published:** 2012-11-27

**Authors:** Inger Brandsma, Dik C Gent

**Affiliations:** 1 Department of Genetics, Erasmus MC, University Medical Center Rotterdam, Rotterdam, The Netherlands

**Keywords:** DSB repair, HR, NHEJ, DNA repair assays, PARP inhibitors

## Abstract

Proper repair of DNA double strand breaks (DSBs) is vital for the preservation of genomic integrity. There are two main pathways that repair DSBs, Homologous recombination (HR) and Non-homologous end-joining (NHEJ). HR is restricted to the S and G2 phases of the cell cycle due to the requirement for the sister chromatid as a template, while NHEJ is active throughout the cell cycle and does not rely on a template. The balance between both pathways is essential for genome stability and numerous assays have been developed to measure the efficiency of the two pathways. Several proteins are known to affect the balance between HR and NHEJ and the complexity of the break also plays a role. In this review we describe several repair assays to determine the efficiencies of both pathways. We discuss how disturbance of the balance between HR and NHEJ can lead to disease, but also how it can be exploited for cancer treatment.

## Introduction

Genomic integrity and faithful replication are essential to prevent mutations and chromosomal rearrangements, which may otherwise lead to diseases and in some cases even death. DNA damage is generated by several different genotoxic agents such as reactive oxygen species, UV light from the sun and mutagenic chemicals [1]. These agents cause many types of DNA damage, ranging from base damage to double strand breaks (DSBs). To protect the genome from the deleterious effects of these lesions, several mechanisms have evolved that detect and repair DNA damage. Together with mechanisms that regulate cell cycle progression and cell death pathways this is known as the DNA damage response (DDR).

In this review we concentrate on DSBs, which are among the most cytotoxic types of DNA damage. The therapeutic effect of several commonly used cancer treatment modalities, such as ionizing radiation and the chemotherapeutic doxorubicin, are based on the cell-killing effect of DSBs. However, DSBs are also the initiating lesion of disease-causing chromosomal translocations in cancer. Therefore, it is important to understand the intricate regulation of the DDR upon DSB formation. We mainly concentrate on the two main DSB repair pathways, Non-homologous end joining (NHEJ) and Homologous recombination (HR), with a special emphasis on the balance between both repair mechanisms in health and disease.

### NHEJ

**Figure 1:**
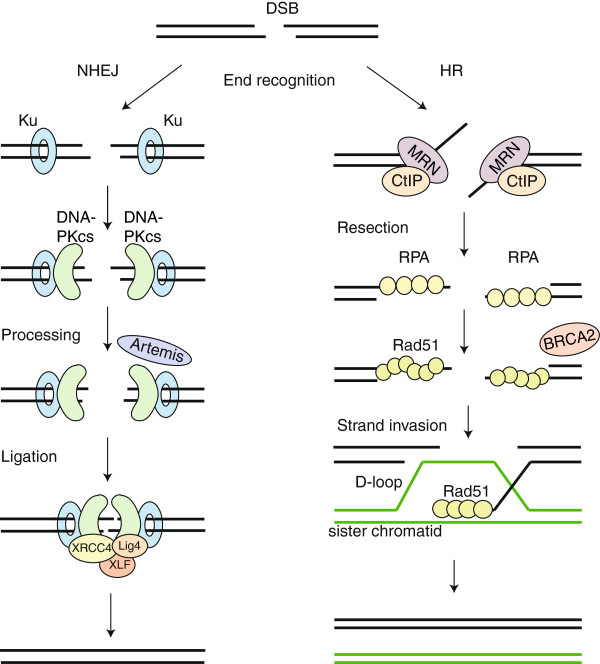
**HR and NHEJ.** NHEJ) NHEJ starts with recognition of the DNA ends by the Ku70/80 heterodimer, which recruits DNA-PKcs. If the ends are incompatible, nucleases such as Artemis can trim the ends. The XRCC4-DNA Ligase IV-XLF ligation complex seals the break. HR) The MRN-CtIP-complex starts resection on the breaks to generate single stranded DNA (ssDNA). After resection the break can no longer be repaired by NHEJ. The ssDNA is first coated by RPA, which is subsequently replaced by Rad51 with the help of BRCA2. These Rad51 nucleoprotein filaments mediate strand invasion on the homologous template. Extension of the D-loop and capture of the second end lead to repair.

NHEJ is a relatively simple DSB repair pathway (Figure 1). Both ends of the break are first bound by the Ku70/Ku80 heterodimer, which then recruits the catalytic subunit of the DNA dependent protein kinase (DNA-PKcs) [2]. If necessary, the ends can be trimmed by nucleases (such as Artemis) or filled in by DNA polymerases (such as Polμ or Polλ) to create compatible ends [3]. Finally, the ligation complex, consisting of DNA ligase IV, X-ray cross-complementation group 4 (XRCC4) and Xrcc4 like factor (XLF)/Cernunnos ligates the ends [4,5]. NHEJ can take place throughout the cell cycle. For an extensive review on NHEJ see [3].

### HR

HR uses a sequence similar or identical to the broken DNA as a template for accurate repair. The sister chromatid is used as an identical template in the S and G2 phases of the cell cycle, when the DNA has been replicated. HR is restricted to these cell cycle phases in higher eukaryotes to prevent recombination between (repetitive) non-identical sequences. Spurious HR can lead to loss of heterozygosity (when HR takes place between paternal and maternal chromosomes) or insertions/deletion (when repeats are not aligned properly).

The HR pathway starts with resection of the broken DNA ends (Figure 1) by the MRN-complex [6,7], together with CtBP-interacting protein (CtIP) [8,9] and other exonucleases, generating 3’-single stranded DNA (ssDNA) [9,10]. The ssDNA tail is coated by Replication protein A (RPA) to remove secondary structure [11]. Subsequently, BRCA2 mediates the replacement of RPA by RAD51, to form a nucleoprotein filament that searches for the homologous sequence on the sister chromatid. After strand invasion, catalyzed by RAD51 and many other proteins, the DNA end is extended using the intact sequence as a template. After restoration of any lost sequence information, the second end of the broken DNA is captured and the junctions are resolved to give a precisely repaired DSB [12]. This resolution step can be accomplished via formation of two Holiday junctions, which are subsequently resolved to give crossover or non-crossover products (the double Holiday junction model). An alternative HR model, the synthesis dependent strand annealing (SDSA) model, does not involve Holiday junctions and results in non-crossover products only [13].

### Foci

Microscopically, DSBs can be visualized as local spots of repair protein accumulation (also called foci) in the nucleus. For example, histone H2AX is phosphorylated locally around the DSB and 53BP1, RPA and RAD51 accumulate in foci after ionizing radiation. Changes in the number of foci per nucleus in time can be quantified to analyze the dynamics of DNA repair [14]. Not all repair proteins accumulate in sufficient numbers to form foci. For example Ku70/80 does not form foci, although it is recruited to DNA damage [15,16].

### Alternative DSB repair pathways

In addition to classical HR, several subpathways result in slightly different products. For example the single strand annealing (SSA) pathway uses directly repeated stretches of homology to repair DSBs. After resection of the break (as described above for HR) complementary stretches in the ssDNA anneal and the intervening sequence and one of the repeats is deleted [17]. Since HR and SSA use the same substrate, these pathways compete when repeats are present on both sides of the break and SSA should be suppressed to prevent its mutagenic effect.

Alternative end-joining pathways can also join DSBs in an error-prone manner, especially when classical NHEJ is impaired by deletion of essential components. The genetics of this pathway are not well defined and there may even be several alternative end-joining pathways. A dependence on DNA ligase III, Xrcc1 and PARP1 has been found in genetic assays [18,19]. However, in another assay the repair of I-SceI induced DSBs in XRCC4-deficient pro-B cell lines did not require Xrcc1 [20]. Alternative pathways show increased DSB joining using microhomologies (stretches of 1–6 bp of direct repeat at the junction), possibly to stabilize the synapsed ends [3].

Although these alternative DSB repair pathways can work in specific experimental settings, they probably do not play a major role in repair of most DSBs in wild type cells. Therefore this review will focus on the balance between the classical forms of HR and NHEJ.

### Repair assays

To study the balance between HR and NHEJ, one would ideally measure both types of repair at the same time using a defined chromosomal site. Unfortunately such an assay is not yet available. There are, however, many assays to measure HR and NHEJ separately. A good understanding of these assays is indispensable for correct interpretation of the results obtained using these different approaches. We therefore review the major assay systems and discuss their merits and drawbacks.

### Assays to measure NHEJ

**Figure 2:**
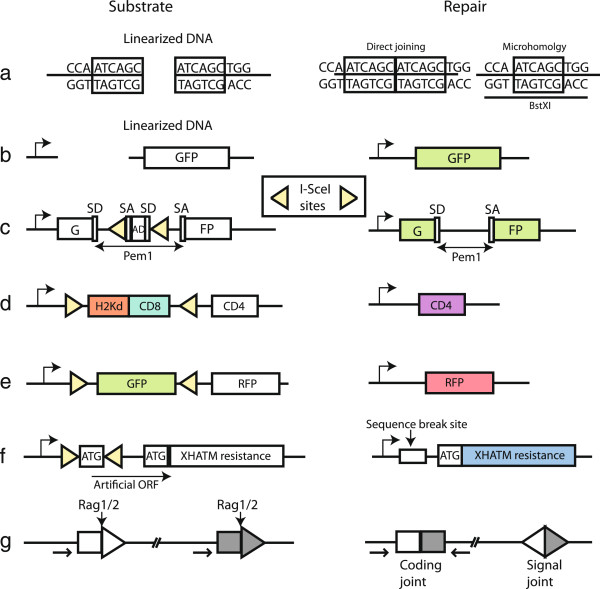
**NHEJ repair assays. a**) Linear plasmid DNA with 6 bp repeats at the ends is joined after transfection. The joints are amplified by PCR and digested using BstXI to distinguish between direct repair and microhomology mediated repair [21]. **b**) Repair of linearized plasmid DNA results in restoration of GFP expression. **c**) Cleavage by I-SceI and subsequent repair lead to loss of the middle splice donor and acceptor sites (SD and SA) and the adenoviral exon (AD), resulting in the expression of active GFP [22]. **d**) H2Kd fused to CD8 is expressed from the intact substrate. Repair of the oppositely oriented I-SceI breaks results in loss of H2Kd-CD8 and allows expression of CD4 [23]. **e**) Similar to d), the intact substrate expresses GFP, while the repaired substrate allows expression of RFP and loses GFP expression [24]. **f**) Between the opposite I-SceI sites, a translation start site is located, preventing translation of the XHATM resistance gene. Repair of the I-SceI breaks and loss of the intervening ATG results in XHATM resistance. The sequence around the breaks can be sequenced to monitor loss of nucleotides [25]. **g**) V(D)J recombination assay. Cleavage by the Rag1/2 endonuclease at the recombination signal sequences induces inversion of the intervening sequence. Small arrows indicate location of PCR primers to amplify joints [21].

NHEJ can be measured in many different ways. The simplest version is transfection of linearized DNA into wild type and mutant cells. Joining of the ends can be monitored by cloning out individual plasmids or PCR amplification followed by sequencing or digestion of the junction [21] (Figure 2a). Recircularization can also be monitored by following the restoration of expression of a reporter gene, such as an antibiotic resistance gene or a fluorescent marker (Figure 2b). A major disadvantage of these assays is that the linear DNA is extrachromosomal and NHEJ cannot be measured in the normal context of chromatin. However, it is a simple assay that can monitor decreased activity of the core NHEJ machinery as a shift towards microhomology use at the newly formed junctions.

Another type of NHEJ assays uses two I-SceI restriction sites. These sites can be in the same or opposite orientation, generating compatible or incompatible ends, respectively. These constructs are generally integrated into the genome. The general theme of all these assays is restoration of expression of a marker gene, in some cases accompanied by inactivation of another gene. Mao et al. (Figure 2c) interrupted the GFP gene with an intron containing an adenoviral exon (AD) flanked by two I-SceI sites. Repair of the two I-SceI induced DSBs leads to loss of the intervening exon and expression of functional GFP [22]. Guirouilh-Barbat et al. developed a similar assay (Figure 2d) with compatible or incompatible I-SceI sites, but they used surface antigens as a read-out for repair [23]. Coleman and Greenberg also used a comparable assay (Figure 2e) with GFP between the I-SceI sites and RFP downstream, resulting in loss of GFP and expression of RFP after repair of the I-SceI induced DSBs [24]. In these assays with a double I-SceI site it is also possible to sequence the joints and to determine the loss of nucleotides around the breaks (Figure 2f) [25].

A disadvantage of these assays using I-SceI restriction sites is that the individual I-SceI break has compatible ends and can recreate an I-SceI site if it is repaired precisely by NHEJ. Therefore, several cycles of cleavage and repair can happen before the site is lost due to inaccurate repair and these assays cannot measure the NHEJ efficiency accurately. However, sequencing of the junctions can provide interesting information about imprecise end-joining events. To avoid the cut-and-paste cycle problem of the I-SceI sites, some assays use transposon excision to create a break. Repair of transposon-induced DSBs can reveal details of efficiency as well as precision of NHEJ [26,27]. In principle, transposons would also be useful to study HR, although their DSB formation efficiency is generally lower than endonucleases.

The immune system depends on end-joining for V(D)J and class switch recombination (CSR). Pan-Hammarstrom and colleagues studied CSR by PCR amplification and sequencing of the junctions in normal individuals and patients. They found that patients with mutations in NHEJ components, such as DNA ligase IV, showed an increased dependence on longer microhomology stretches at the junctions [28]. An advantage of this assay is that repair is measured on endogenous substrates. However, it is not clear whether these loci are representative for other types of DSBs.

V(D)J recombination also depends on NHEJ factors to repair the breaks induced by the Rag1 and Rag2 proteins. This type of repair can be assayed using a specific repair substrate containing Recombination Signal Sequences, the recognition sites for Rag1 and Rag2 (Figure 2g) [21]. The V(D)J recombination assay gives a clear phenotype for defects in proteins involved in DNA end-processing, such as the Artemis nuclease [29]. The major disadvantage of these types of assays is the special nature of the DSBs formed by the RAG proteins, which may shuttle the breaks towards NHEJ [30].

### Assays to measure HR

**Figure 3:**
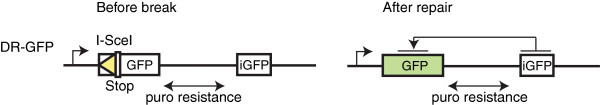
**HR Assay.** The 5’ GFP is inactivated by several in frame stop codons and contains an I-SceI site. A downstream truncated GFP, lacking the I-SceI sites and stops, serves as a template. Accurate repair via HR results in GFP expression.

The most commonly used assay to measure HR is the DR-GFP assay developed by Pierce and Jasin [31] (Figure 3). The reporter construct can be inserted by gene targeting or random integration. It contains two GFP sequences separated by a selection marker. The 5’ GFP sequence is inactivated by an I-SceI site and internal stop codons, preventing GFP expression. The 3’ truncated GFP serves as a template for repair after DSB induction by I-SceI. Repair of the break by gene conversion using the downstream GFP sequence leads to restoration of the GFP gene and the percentage of GFP expressing cells can be determined by FACS analysis.

This HR assay has been used successfully to characterize defects in various (repair) mutant genetic backgrounds. An important advantage of this HR assay is that it measures repair using a chromatinized reporter construct in the chromosome. However, the template for repair is downstream of the break, whereas the normal template for HR is the equivalent position on the sister chromatid. Furthermore, the I-SceI site can be subject to several cycles of cleavage and repair by precise NHEJ or restoration of the sequence using the sister chromatid as a repair template, which leaves a high degree of uncertainty about the relative levels of HR and NHEJ.

Expression of I-SceI is usually induced by transfection of an expression plasmid into an asynchronously growing cell population. This creates DSBs in the reporter substrate throughout the cell cycle, whereas HR only takes place in the S and G2 phases. To overcome this problem, Hartlerode et al. developed an I-SceI fusion protein that is drug-activatable. Enriching cells in a certain phase of the cell cycle then allows restricted activation of I-SceI [32].

HR can also be estimated by scoring sister chromatid exchanges (SCEs). In this assays a nucleotide analog is added in the first cell cycle to allow incorporation into the newly synthesized strand in S phase. After a second replication round, only one of the sister chromatids is labeled, which allows visualization of recombination between the sister chromatids in metaphase spreads by staining for the incorporated nucleotide analog. SCEs can be formed in S phase during the repair of collapsed replication forks as well as in G2 phase at two-ended DSBs [33].

A completely different method to assess the efficiency of DSB repair is monitoring the disappearance of γH2AX foci. These foci form within a few minutes after DSB formation and disappear slowly as repair takes place. By comparing the kinetics of several known HR and NHEJ mutants, the efficiency and likely repair pathway can be determined. For a review on the advantages and potential pitfalls of this assay, see [34].

As a more sophisticated approach, the formation and disappearance of 53BP1-YFP and Rad52-Cherry foci has been followed to estimate the use of HR and NHEJ in single cells throughout the cell cycle [35]. Karanam et al. found that there is a gradual increase in HR at the beginning of S phase. The number of Rad52 foci increases till mid S phase and then decreases towards the end of S phase. In G2, very few Rad52 foci were observed, showing that HR is not the predominant pathway in G2. This is consistent with data from Beucher et al., who demonstrated that NHEJ repairs approximately 85% of all IR-induced DSBs in G2, as measured by γH2AX foci kinetics [36].

### Balancing HR and NHEJ

The presence of large numbers of highly repetitive sequences in the DNA of higher eukaryotes makes HR between sequences other than sister chromatids prone to misalignment of the homologous sequences. Therefore, HR generally dominates in organisms with a small genome (with low abundance of repetitive sequences), whereas mammals mainly rely on NHEJ for DSB repair [35,37]. However, even in highly complex genomes, HR is used as the preferred DSB repair mechanism to deal with DSBs that are formed during replication. This necessitates intricate control mechanisms to prevent access of the wrong repair pathway to the DSB.

### Resection & cell cycle

HR can only safely be used to repair breaks in the S and G2 phases of the cell cycle. The first mechanism to regulate this depends on S/G2 specific cyclin dependent kinases (CDKs). DNA end resection requires phosphorylation of CtIP on a CDK consensus sequence [9,38,39]. Proteasome-mediated degradation of the CtIP protein in G1 [40] adds an additional layer of regulation at the resection step.

CDK1/CyclinB also phosphorylates the NBS1 component of the MRN complex on Serine 432 during the S, G2 and M phases, which is required for resection and efficient HR. However, IR sensitivity was not affected in the Ser432Ala NBS1 mutant, consistent with the notion that NHEJ is the major DSB repair pathway in mammals [41].

Although activation of HR proteins in a cell cycle dependent manner helps to restrict their activity, it is insufficient to ensure safe use of HR. While replication is ongoing in the S phase, parts of the genome have not yet been replicated and recombination of these parts should be avoided to prevent loss of heterozygosity and non-allelic recombination. Therefore, another layer of regulation is provided by the structural maintenance of chromosomes (SMC) proteins such as Cohesin, Condensin and SMC5/6: they are able to confine repair to the sister chromatid and prevent HR between other sequences [42,43].

### Complexity of the break

Whether HR or NHEJ is used also depends on DSB complexity. This phenomenon has been studied in detail in the G2 phase of the cell cycle, when both HR and NHEJ contribute to DSB repair. Treatment of cells with the topoisomerase II inhibitor Etoposide results in breaks with a 4 bp 5’-overhang with covalently attached protein [44]. The large majority of these breaks are repaired rapidly by NHEJ. The remaining 10% of the Etoposide induced breaks is repaired with slow kinetics via HR [45]. High linear energy transfer (LET) carbon ions, on the other hand, induce highly complex clusters of DSBs and other types of DNA damage [46], because this type of radiation causes a high number of ionizations in a small volume. These breaks are frequently resected and their repair takes place via HR with slow kinetics [45]. From the breaks induced by low LET ionizing irradiation (IR), which causes less complex DSBs, only 20-30% is resected and their repair is much less dependent on HR [36,45].

The chromatin structure around the DSB affects repair as well. Breaks in heterochromatin are repaired more slowly than breaks in euchromatin [47] probably because euchromatin is more easily accessible for repair and requires less or no remodeling. Repair of breaks in heterochromatin requires ATM [47]. ATM phosphorylates transcriptional corepressor Krüppel-associated box (KRAB)-associated protein (KAP)-1 [48], which disrupts the interaction between Kap-1 and CHD3 [49]. CHD3 is an ATP-dependent nucleosome remodeling enzyme and its dispersion allows chromatin relaxation, facilitating DSB repair in heterochromatin. Furthermore, the ATP-dependent chromatin remodeler SMARCAD1 can also be recruited to sites of DNA damage where it facilitates resection [50].

### Genetic factors influencing pathway choice

The core HR and NHEJ machineries have been conserved from yeast to mammals [51]. However, several genes have been added to optimize or regulate both pathways in higher eukaryotes. For example, NHEJ has acquired DNA-PK_CS_ and HR added several RAD51 paralogs. Furthermore, several additional genes in higher eukaryotes regulate DSB repair pathway choice without direct participation in the catalytic steps of the repair reaction.

### 53BP1

The p53 binding protein 1 (53BP1) is recruited to DSBs, where it has functions in cell cycle checkpoint maintenance and double strand break repair [52]. The fast phase in DSB repair is normal in the absence of 53BP1, but repair of breaks in heterochromatin is severely impaired, probably as a result of impaired Kap1 phosphorylation [49,53].

A deeper understanding of the 53BP1 function has been gained from studies in the immune system. During class switch recombination, highly repetitive DNA segments are recombined to generate the different classes of antibodies. DSBs generated during this recombination reaction can be repaired via NHEJ or alternative end joining. In the absence of 53BP1, resection increases and microhomology mediated alternative end-joining takes over from classical NHEJ [54]. In V(D)J recombination, Variable (V), Diversity (D) and Joining (J) segments are recombined to create a large variety of functional coding sequences for immunoglobulins and T-cell receptors. DSBs created by Rag1/2 are repaired via NHEJ. 53BP1 prevents extensive degradation and it promotes synapsis of DNA ends and stabilizes long-range interactions, not only between breaks created during V(D)J recombination [55], but also between deprotected telomeres [56].

### BRCA1 and associated protein

In contrast to the NHEJ promoting effect of 53BP1, the tumor suppressor BRCA1 is required for efficient HR [57] and formation of Rad51 foci after DSB induction [58]. BRCA1 is an E3-ubiquitin ligase that forms a complex with the E2 enzyme BARD1 via its RING domain. This interaction is required for the ligase activity, as well as protein stability and nuclear localization [59]. Although several RING domain mutations have been found in patients, it is currently unknown how the HR defect is related to the E3-ligase function and BARD1 interaction. Drost et al. recently showed that the ring domain is necessary for tumor suppression, but not required for the development of resistance to chemotherapeutics. Tumors with a C61G mutation in the RING domain rapidly develop resistance to platinum drugs and the PARP inhibitor Olaparib, while retaining this mutation [60].

In addition to its function as a ubiquitin ligase, BRCA1 may also function as a scaffold protein that associates with many interaction partners, such as Abraxas, BACH1 and BRCA2/PALB2 [59]. For efficient resection of DNA ends, its interaction with CtIP and the MRN complex is probably important [39,61]. BRCA1 also interacts with RAP80 and the BRCA1/RAP80 complex is recruited to ubiquitylated chromatin around DSBs [62-64]. In contrast to the BRCA1 interactions described above, the RAP80-BRCA1 interaction decreased HR: depletion of Rap80 stimulated recruitment of CtIP and Mre11 and thereby resection [24]. The BRCA1 interactions with CtIP and RAP80 are mutually exclusive, indicating that competition for this BRCA1 binding site affects resection and thereby pathway choice. For replication-associated breaks, BRCA1 clearly tips the balance towards HR.

### Genetic interactions of BRCA1 and 53BP1

Recently, some unexpected genetic interactions between BRCA1 and 53BP1 shed new light on their function in balancing DSB repair pathways. Deletion of BRCA1 causes embryonic lethality, but this can be rescued by deletion of 53BP1 [65]. Unexpectedly, deletion of 53BP1 also restored HR and Rad51 foci formation in BRCA1 deficient cells [66,67], implying that both factors influence HR in opposite directions and that inactivation of both genes largely restores the balance. Inactivation of 53BP1 in BRCA1^−/−^ ES cells led to an increase in both nucleolytic DNA end processing and RPA phosphorylation [67]. ATM inhibition in BRCA1 ^−/−^ 53BP1^−/−^ cells reduced RPA phosphorylation and Rad51 foci formation, indicating that ATM-dependent resection allows partial restoration of HR [67].

**Figure 4:**
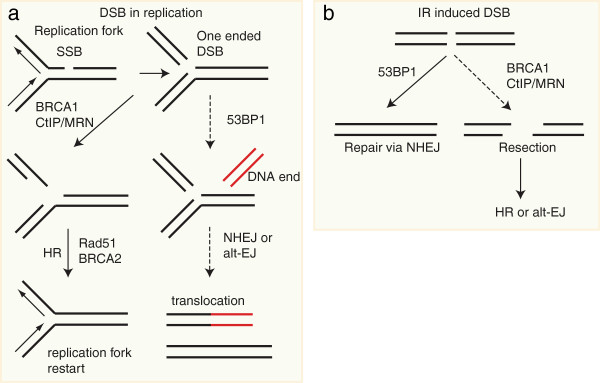
**BRCA1 and 53BP1 in DSB repair. a**) Repair of replication associated breaks requires HR. 53BP1 blocks resection of the one-ended break in BRCA1 deficient cells, preventing repair via HR. The breaks are either left unrepaired or repaired via NHEJ using other random DNA ends, which leads to chromosomal rearrangements and genomic instability. In the absence of 53BP1, resection of the DNA ends can take place, allowing faithful repair via HR. **b**) IR induced two ended DSBs are mainly repaired via NHEJ, however part of the breaks is repaired via HR or alternative end-joining (alt-EJ). Repair via HR or alt-EJ increases when classical NHEJ is impaired by a mutation in one of the core NHEJ genes or 53BP1.

Figure 4 presents a plausible model to accommodate these findings. One-ended DSBs that are formed during replication require BRCA1 to stimulate resection. In BRCA1 deficient cells, 53BP1 prevents resection of DNA ends, leading to aberrant diversion of breaks to NHEJ. This creates dead-end products (if only one DNA end is present) or inappropriate joining to distant sequences causing chromosomal translocations (if a DNA end combines with an unrelated other DNA end). Two-ended DSBs, on the other hand, require 53BP1 to limit resection and allow efficient NHEJ. Overactive resection in 53BP1^−/−^ cells may result in aberrant HR reactions (such as single-strand annealing) or alternative end-joining pathways, creating microhomology-mediated translocations and/or junctions with excessive deletions [68,69].

Further insight into the role of BRCA1 and 53BP1 in repair pathway choice was recently obtained using super resolution microscopy of IR induced foci (IRIF). The core of the focus contained mainly 53BP1 molecules in the G1 phase of the cell cycle, probably representing repair via NHEJ. In S phase, however, the core of the IRIF was filled with BRCA1 and 53BP1 formed a ring around this core, suggesting that BRCA1 physically excludes 53BP1 from the break to allow repair via HR [70].

BRCA1 deficient cells are exquisitely sensitive to PARP inhibitors, which inhibit single strand break repair [71,72]. The rationale for this observation is that replication of DNA with single strand breaks results in formation of single DNA ends, which require HR for their repair (Figure 4). As described above, deletion of 53BP1 in BRCA1 deficient cells rescues embryonic lethality. However, loss of 53BP1 also leads to resistance to PARP inhibition [66,73]. In the BRCA1-deficient cells that have also lost 53BP1, the number of chromosome and chromatid breaks is decreased and checkpoint activation is diminished compared to cells that are only BRCA1 deficient [66], suggesting that the regained HR capacity in these cells is largely sufficient to restore genomic stability. A subset BRCA1 and BRCA2 mutant tumors shows loss of 53BP1, indicating that therapy resistance via loss of 53BP1 may be clinically relevant [66].

### Ubiquitylation and sumoylation

Ubiquitin and the small ubiquitin-like modifier (SUMO) are small polypeptides that can be attached to proteins as a posttranslational modification. After activation of ubiquitin or SUMO by an E1 enzyme, they are transferred to an E2 conjugating enzyme. With the help of an ubiquitin (or SUMO) ligase (E3) the modification is attached to the substrate. Deubiquitylating enzymes (DUBs) can reverse the ubiquitin modification.

Many proteins involved in the DDR can be ubiquitylated or sumoylated [2,74-76]. For the sake of simplicity, we will focus on one part of the DDR signaling cascade as an example. Upon DSB formation, histone H2AX is phosphorylated by ATM or DNA-PK. MDC1 is recruited to this phosphorylated histone (γH2AX) and is in turn phosphorylated by ATM. This attracts the E3 ligase RNF8 which ubiquitylates H2A and H2AX. Subsequent action of the E3 ligase RNF168 leads to more extensive ubiquitylation of the chromatin around the break, creating a recruitment platform for many other repair proteins, including 53BP1 and BRCA1 [77]. These ubiquitylation events are also required for phospho-KAP-1 foci formation and thereby chromatin relaxation at sites of damage [53].

In addition to an effect on recruitment of repair proteins, ubiquitylation can also affect release of proteins from the lesion. The transient binding of Ku at DNA ends affects pathway choice. Ku binds in all phases of the cell cycle and must be removed to allow resection [78,79]. This removal can be facilitated via ubiquitlyation of Ku by the E3 ligase RNF8 and an unknown E2 conjugating enzyme, leading to proteasome-dependent Ku degradation [80]. Since Ubiquitylation is a very abundant modification on DDR proteins, it is likely that more modifications affecting pathway choice will be discovered in the future.

## Concluding remarks

A unifying model for DSB repair pathway choice should take into account that NHEJ is relatively fast, while resection is a slow process that probably creates a point of no return. Therefore, it is to be expected that NHEJ initially tries to repair all DSBs and only if this repair pathway fails to repair the lesion, the chance that resection takes place increases over time, necessitating repair via HR. This is consistent with the observation that the binding of the Ku heterodimer to DNA ends is a very fast process, but the assembly of end-joining complexes is dynamic and may in the long run give way to proteins mediating resection if they are active [16]. This means that initiation of HR will mainly be restricted to the S and G2 phases of the cell cycle, when CtIP is active. Indeed, a subfraction of DSBs in G2 requires BRCA2 for their repair, but knock-down of both CtIP and BRCA2 alleviates this repair defect [45], suggesting that avoiding resection prevents HR and allows repair of these DSBs by NHEJ. Replication associated breaks, on the other hand, should be channeled to HR, which is the only pathway that can restart a replication fork from a single broken DNA end.

The study of the balance between HR and NHEJ is important for the prediction of treatment responses upon inhibition of these pathways in various genetic backgrounds. Combined treatments might backfire when the balance is tipped the wrong way. For example, the treatment of BRCA1 and BRCA2 deficient cells is most effective when NHEJ is functional, whereas impaired NHEJ prevents lethal genomic instability and cytotoxicity, which counteracts the effect of PARP inhibitors in HR deficient cells [73,81].

The balance between HR and NHEJ is heavily regulated, but the wiring and hierarchy of this regulatory network is still incompletely understood. Development of targeted therapies using DNA damage response defects requires a much more detailed knowledge of the precise network of the cellular responses to DNA damaging treatments. It is to be expected that new assay systems will be developed and that a flurry of novel combinations of chemical inhibitors and genetic defects will increase our understanding of these processes in the near future. This knowledge will then be an invaluable source for developing new targeted therapies for tumors with DNA damage response defects, which should yield more specific and effective therapeutic approaches to combat cancer. Novel tools to characterize tumor-specific (DNA repair gene) mutations, such as whole genome sequencing approaches, should then bring truly personalized medicine for cancer treatment within reach.

## Competing interests

The authors declare that hey have no competing interests.

## Authors’ contributions

IB wrote the first draft. IB and DvG wrote the final text. Both authors read and approved the final manuscript.
